# Routine thyroglobulin, neck ultrasound and physical examination in the routine follow up of patients with differentiated thyroid cancer—Where is the evidence?

**DOI:** 10.1007/s12020-018-1720-3

**Published:** 2018-08-20

**Authors:** Jessica L. Gray, Gautam Singh, Lesley Uttley, Saba P. Balasubramanian

**Affiliations:** 1 Department of Oncology and Metabolism, University of Sheffield, Sheffield, UK; 2 Endocrine Surgery Unit, Directorate of General Surgery, Sheffield Teaching Hospitals, Sheffield, UK; 3 School of Health and Related Research, University of Sheffield, Sheffield, UK

**Keywords:** Thyroid cancer, Recurrence, Thyroglobulin, Ultrasonography, Palpation, Surveillance

## Abstract

**Purpose:**

Patients with differentiated thyroid cancer (DTC) typically have a favourable prognosis and recurrence as late as 45 years after diagnosis has been reported. International clinical guidelines for monitoring recommend routine thyroglobulin, ultrasound and physical examination for the detection of recurrence. The aim of this review was to systematically review whether routine monitoring using thyroglobulin (Tg), neck ultrasound and physical examination for recurrence in differentiated thyroid cancer patients is effective in improving patient survival and/or quality of life.

**Methods:**

Primary studies were retrieved via a comprehensive search of three electronic bibliographic databases (PubMed, Web of Science Core Collection and Cochrane Library) without time restriction. Eligible studies must have reported on disease-free patients with DTC subject to long-term routine surveillance. The primary and secondary outcomes of interest were overall survival (or other survival parameters) and quality of life, respectively.

**Results:**

Literature searches yielded 5529 citations, which were screened by two reviewers. 241 full texts were retrieved. No randomised controlled trials or two-arm cohort studies on the effectiveness of any of the three specified interventions were identified. However, three ‘single-arm’ studies reporting long-term follow-up outcomes in patients undergoing regular surveillance were identified and appraised.

**Conclusions:**

This review highlights a lack of empirical evidence to support current use of routine surveillance in DTC. Although early detection is possible, routine surveillance may lead to unnecessary intervention.

## Introduction

The vast majority of thyroid malignancy is differentiated thyroid cancer (DTC), originating from thyroid follicular epithelium. DTC is comprised of papillary thyroid cancer (PTC) in 85–90% of instances and follicular thyroid cancer (FTC) in 5–10% [1]. The incidence of thyroid cancer has risen over the past few decades, largely driven by an increase in PTC [2]. An associated decrease in mortality rates [3] suggests that clinically insignificant PTC is increasingly being identified.

DTC is usually indolent and often found incidentally [4]. The overall prognosis at 10 years is 90–95%. Patients who are disease free following treatment have a life expectancy similar to the general population [4, 5]. However, 5–20% of patients develop local recurrence and over 10% develop distant metastases; the risk increasing with age at diagnosis [6]. Up to two-thirds of relapses can be detected within the first decade by serum thyroglobulin (Tg) and imaging, but some relapses are observed as late as 45 years [7, 8]. The late recurrences reported in earlier studies may be influenced by follow-up protocols used in these populations that may be different to current regimes in terms of imaging modalities used and length of follow up; but this data has been used as the basis for current practice in several countries to routinely monitor patients for life. Clinical guidelines produced by the American Thyroid Association (ATA), the British Thyroid Association (BTA) and the European Society of Medical Oncology (ESMO) [6, 9, 10] recommend differing regimes involving routine measurements of serum Tg, neck ultrasound (US) and physical examination (PE) to detect recurrence.

Tg is a dimeric glycoprotein released by normal follicular tissue and DTC [11]. Tg detection after thyroidectomy suggests either residual thyroid tissue or persistent or recurrent cancer [12]. Although recommended as a routine test for monitoring recurrence in DTC, there are concerns regarding assay sensitivity and inter-assay variation [9, 13]. False-negatives results may occur due to interference with anti-Tg antibodies (TgAb) in up to 30% of patients from saturation and Tg-negative tumours [13–15]. Tg measurement under TSH stimulation enhances test sensitivity [16]. More significantly, the trend in serial Tg levels is more accurate in detecting cancer recurrence [17].

Since the 1960s, US has transformed thyroid cancer management in the detection of recurrence by guiding biopsies and mapping disease before surgery [18]. US may be more sensitive than serum Tg measurements or radioiodine whole body scans (WBS) [19]. However, detection of lesions as small as 2–3 mm has increased detection of subclinical recurrent disease [20]. At these sizes, US does not distinguish between residual thyroid tissue and malignant disease [9, 20]. This increases the risk of false-positive US, unnecessary biopsy and leads to increased anxiety in patients, particularly in ‘low risk’ disease [18, 21].

PE identifies palpable thyroid nodules in around 90% of symptomatic DTC patients. However, only 5% of all patients presenting with thyroid nodules have thyroid cancer [10]. For initial diagnosis, clinicians evaluate the likelihood of malignancy based on consistency, history of rapid growth and fixity; supported by other features including voice change, compressive symptoms and palpable lymphadenopathy [6]. In the absence of worrying features on examination and imaging, some palpable lumps may simply be observed in the pre-diagnosis setting. However, due to the risk of recurrence, any palpable mass detected during follow-up of thyroid cancer patients is treated with suspicion, however, not all recurrences are palpable. The sensitivity of examination varies with clinician experience and centre volume and is lower than radioiodine WBS, serum Tg measurements and US scans [19]. As with US, its utility is limited to the detection of local recurrence and not distant metastasis.

Given the uncertainties highlighted above, it is unsurprising that clinical practice and guidelines from various organisations vary significantly, as shown in Table 1.

**Table 1:** The American Thyroid Association (ATA), British Thyroid Association (BTA) and European Society of Medical Oncology (ESMO) recommendations on routine monitoring of patients with DTC and the ‘self-reported’ basis of these recommendations

ATA	Follow-up of low-risk patients should include PE ⇒ Weak recommendation ⇒ Low-quality evidence	Tg (and TgAb) every 6–24 months dependent on ‘risk’ ⇒ Strong recommendation ⇒ Moderate to low-quality evidence	US every 6–24 months dependent on ‘risk’ ⇒ Strong recommendation ⇒ Moderate to low-quality evidence
BTA	Follow-up should include PE ⇒ Weak recommendation ⇒ Expert opinion	Tg (and TgAb) no more frequently than 3 monthly ⇒ Weak recommendation ⇒ Expert opinion	No recommendation
ESMO	Follow-up should include PE ⇒ No grading of evidence or recommendation	Tg annually ⇒ No grading of evidence or recommendation	US annually ⇒ No grading of evidence or recommendation

Based on the ATA risk stratification (Table 2), low-risk patients may be monitored annually after the initial 6–12 monthly follow-ups and high-risk patients are to be monitored 6–12 monthly for as long as deemed necessary [9]. This practice is ‘strongly recommended’, but based on moderate and low quality evidence; where there was either ‘minor’ or ‘serious’ concern regarding the ‘internal validity or external generalizability of the results’ [9]. Also, the guidelines cite studies focussing on the technical aspects of interventions (i.e. different Tg assay sensitivities, anti-Tg antibody interference, US criteria for malignancy) and surrogate outcomes (such as assessing radioiodine ablation (RIA) success); but not on clinical outcomes such as quality of life or survival.

**Table 2:** American Thyroid Association (ATA) and European Society of Medical Oncology (ESMO) risk stratification criteria for differentiated thyroid cancer

ATA [9]		
Low risk	Intermediate risk	High risk
No local/distant metastases	Microscopic local invasion	Macroscopic invasion
All macroscopic tumour resected	Cervical LNM OR Positive post-ablation WBS outside the thyroid bed OR Tumour with aggressive histology or vascular invasion	Incomplete resection
No local tumour invasion		Distant metastases
No aggressive histology or vascular invasion		Thyroglobulinaemia that is not proportionate to post-ablative WBS
Negative post-ablation WBS outside the thyroid bed		
**ESMO [** 42 **]**		
**Very low risk**	**Low risk**	**High risk**
Total thyroidectomy	No local/distant metastases	Less than total thyroidectomy
Unifocal carcinoma less than 1 cm with no ETE or LNM	No local tumour invasion No aggressive histology or vascular invasion	Local tumour invasion
		Cervical LNM
		Distant metastases
		Aggressive histology or vascular invasion

*Note*: *WBS* ^131^I whole body scintigraphy, *ETE* extra-thyroidal extension, *LNM* lymph node metastases [40]

The BTA recommends regular PE and Tg no more frequently than three-monthly. Patients who respond well to therapy are seen every 6–12 months. However, this is based on ‘expert opinion’ extrapolated from studies with different primary objectives [6]. The references listed in the guidelines to support the recommendations on monitoring focus primarily on the practicality of Tg [6]. The ESMO recommends annual PE, serum Tg and US for long-term follow-up [10]. The strength of recommendation and the quality of supporting evidence are not clear.

Given the apparent lack of good evidence to support interventions that are currently part of standard care, this systematic review aimed to determine whether there is evidence that routine serum Tg measurement, neck US and PE improve survival and/or quality of life of patients with DTC.

## Methodology

The protocol for this research was registered with the international register of systematic reviews PROSPERO (https://www.crd.york.ac.uk/PROSPERO) in March 2017 (ID: 42017060636).

Medical bibliographic databases including PubMed, Web of Science Core Collection and the Cochrane library were searched from inception until the 11 April 2017 for English-language original articles on this topic. Search terms included thyroid cancer (or neoplasm), recurrence (or relapse or metastasis), Tg, US (or ultrasonography or sonography) and PE (or palpation).

**Fig. 1:**
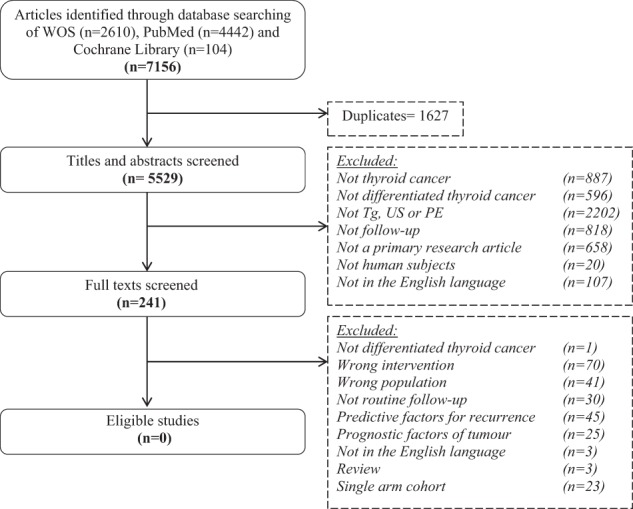
PRISMA flowchart demonstrating the reasons for study exclusion

The review aimed to include randomised controlled trials (RTCs) and non-randomised two-cohort interventional or observational studies evaluating patients undergoing routine serum Tg, neck US or PE (individually or in combination) following treatment for DTC. Single-arm studies with no control group were initially excluded as well as those with historical controls. Patients with poorly differentiated or anaplastic thyroid cancer, as well as those diagnosed with cancer of non-follicular epithelial origin were excluded. Studies must have compared the specific ‘follow up’ intervention to a control cohort that have either not undergone routine surveillance or have undergone a different regime of surveillance. The primary outcome to assess effectiveness was overall survival, measured from diagnosis to death, or other survival parameters, regardless of the length of follow-up. The secondary outcome was the quality of life, measured as defined by the individual studies. The PRISMA flow diagram depicting the identification of studies for the review is shown in Fig. 1.

Two reviewers (J.G. and G.S.) independently screened titles and abstracts generated from the searches described (J.G. and G.S.). Any disagreements were resolved by discussion and the final opinion of the senior author (S.P.B.). A summary of the effects of interventions and tabulation of data on demographics, study design, primary and secondary outcomes, risk of bias and quality of the eligible studies was planned. Meta-analysis of quantitative outcomes was planned but was not possible due the lack of data.

The Cochrane Risk of Bias Tool and the Newcastle–Ottawa Scale (NOS) were selected to assess the quality of RTCs and observational studies, respectively.

Given the lack of trials addressing the question, single arm studies were re-evaluated to determine if data on outcomes, such as survival and quality of life could be collected. Only studies reporting on at least 100 participants and including a follow-up period of at least 5 years were included.

This report was written in accordance to the PRISMA guidance [22]. No external funding was received.

## Results

No eligible RCTs or non-randomised two-arm observational studies that evaluated the effectiveness of routine serum Tg, neck US or PE in improving the survival or quality of life of patients with DTC were identified. The lack of suitable two-arm studies led to a revision in inclusion criteria and subsequent assessment of three single arm cohort studies that fulfilled the eligibility criteria (Table 3).

**Table 3:** Eligible single arm retrospective cohort studies

Author	Population	Risk classification and monitoring protocols	Frequency of monitoring	Follow-up period (average in years)	Survival	Detection of recurrence via Tg
Conrad et al. [23]	343 DTC patients treated with near-total thyroidectomy	130 ‘low risk’ patients: PE + Tg	Annual	6 (parameter not stated)	Overall survival At 10 years: 95% At 20 years: 93%	6/130 had elevated Tg: 4 true positives (detected by WBS) 2 false positives 124 true negatives
		213 ‘high risk’ patients: WBS + Tg + PE → 39/213 showed extra-thyroidal uptake on WBS and received RIA; 7 had persistent disease → 174/213 had uptake suggestive of remnant normal tissue, 135 of which still received RIA; 4 had new extra thyroidal uptake.	WBS at discretion of physician			19/163 patients with ‘physiological’ uptake on WBS had elevated Tg: 10 true positives (detected by WBS) 9 false positives 136 true negatives 8 false negatives (detected by PE) 5/11 patients with ‘persistent’ disease had an elevated Tg.
Lin et al. [15]	847 DTC patients treated with total thyroidectomy and ablation	WBS, CXR+Tg Group A 1 month post-operative Tg < 1 ng/ml Group B 1 month post-operative 1 < Tg < 10 ng/ml Group C 1 month post-operative Tg > 10 ng/ml	6 month intervals	Group A 3.7 (mean) Group B 6.1 (mean) Group C 5.4 (mean)	5-year survival probability: Group A 1.00 Group B 0.992 Group C 0.963 2.36% died of thyroid cancer	Sufficient data not available
Phan et al. (2002)	346 DTC patients treated with thyroidectomy and RIA → 94^a^ patients had undetectable Tg post-ablation with TSH > 30mU/l.	PE, Tg, TgAb, US+MRI^b^ → persistence/recurrence was detected by imaging modalities such as FDG PET or CT (or MRI if Tg was undetectable)	1st year: 3 month intervals 2nd year: 6 month intervals Annually from then on	8 (median)	Not reported	2/94 had elevated Tg: 2 true positives 86 true negatives 6 false negatives (2 persistent) → 3 of the recurrences were TgAb+ve, 1 TgAb −ve

*Abbreviations*: *CT* computed tomography, *DTC* differentiated thyroid cancer, *FDG PET* fluorodeoxyglucose positron emission tomography, *MRI* magnetic resonance imaging, *RIA* radioactive ablation, *Tg* thyroglobulin, *TgAb* thyroglobulin antibodies, *TSH* thyroid stimulating hormone, *US* ultrasound scan, *PE* physical examination, *WBS* ^131^I whole-body scintigraphy

^a^Despite analysis of only 94 participants, the initial number of patients described was >100

^b^MRI was initially performed every 1–2 years and then became less frequent following risk stratification

Conrad et al. [23] stratified 343 participants treated with a near-total thyroidectomy and followed up for an period of 6 (range of 0–20) years. The overall survival was 93% at 20 years and the disease-free survival was 91% and 87% at 10 and 20 years, respectively.

Patients were stratified using the AMES criteria (age, metastasis, multifocality, extent of cancer and size) into low and high risk. 130 low-risk patients were monitored annually by PE and serum Tg. Six demonstrated elevated Tg during follow-up and underwent a radioiodine WBS. Four of six patients showed extra-thyroidal uptake and were treated for recurrence.

The 213 high-risk patients had a post-operative WBS; 39 with extra-thyroidal uptake had RIA and 135 of 174 patients without extra-thyroidal uptake who showed ‘non-physiological uptake’ confined to the thyroid bed received RIA. During the follow-up period, recurrence was diagnosed in 10 of 19 patients with elevated Tg and in eight patients by palpation. Further details regarding site of recurrence or treatment for recurrence were not clear. Overall, only two patients died in the follow-up period from DTC (risk status not clear); one additional patient died of respiratory failure after surgery.

Lin et al. [15] allocated 847 patients treated with a total thyroidectomy and RIA into three groups depending on post-operative Tg within the first month (Group A—1 month Tg of <1 ng/ml; Group B—Tg ≥ 1 ng/ml and <10 ng/ml; Group C—≥ 10 ng/ml) (Table 3). These patients were followed up with 6 monthly WBS, CXR and Tg for a mean of 3.7 ± 0.2 years in Group A, 6.1 ± 0.2 years in Group B and 5.4 ± 0.2 years in Group C. At the end of the study period, 95.8%, 76.4% and 37.1% of patients remained disease-free (defined as a negative WBS and a Tg of <1 ng/ml on follow-up) in groups A, B and C, respectively. The 5-year survival probability was 1.00, 0.992 and 0.963 for Group A, B and C, respectively. There were no deaths in Group A (*n* = 168), six cancer-related deaths in Group B (*n* = 331) and 14 cancer-related deaths in Group C (*n* = 348). Of the cases in Group C, 133 showed detectable Tg levels during follow-up.

Phan et al. [24] analysed 94 of 346 patients who were treated with a near-total thyroidectomy and RIA. These patients had undetectable Tg before ablation and were classified into 30 low-risk patients (<40 years old with no advanced signs of disease) and 64 high-risk patients (>40 years old with late stage or metastatic cancer). The median follow-up period was 8 years (range of 1–17). Eight patients identified to have either persistent (2) or recurrent disease (6) at follow up were all high-risk patients. Three recurrences were Tg negative/antibody positive and detected by palpation of enlarged lymph nodes. The fourth patient identified by PE was Tg positive/antibody negative. A rising Tg level identified the fifth patient with recurrence. The last patient with recurrence was persistently Tg/antibody negative and showed multiple lung lesions on chest x-ray.

## Discussion

A comprehensive search of three electronic databases did not identify any RCTs or non-randomised two-arm studies that evaluated the effectiveness of routine serum Tg, neck US or PE in patients with DTC. Therefore, there is no clear high- quality evidence as to whether routine follow-up improves patients’ quality of life or survival.

It may be that the benefit exists but has not yet been demonstrated. Waiting for symptomatic recurrence may increase treatment morbidity and adversely impact on survival or quality of life. In addition, regular PE by a specialist face-to-face is ‘reassuring’ to the patient [25] and may improve mental well-being.

However, there is potential for harm from unnecessary investigations, treatment-related morbidity, anxiety and/or distress, and potentially unjustified costs to the health service.

Serum Tg has a low positive predictive value (PPV) of <40%, although it increases with the use of serial measurements [17, 26]. Studies have also shown that frequent use of US is more likely to identify false-positive findings than significant disease recurrence [21]. A marked rise in post-operative US surveillance has been associated with an increase in treatment without improvement in disease-specific survival [27].

Reoperation is often used as the definitive treatment for locally recurrent DTC. However, it is recognised that active surveillance of small indolent nodules may avoid unnecessary interventions [9]. The effect of reoperation on survival is unknown, but remission rates can be as low as 19% [28] and morbidity (vocal cord paralysis and hypoparathyroidism) can be significant [29].

**Fig. 2:**
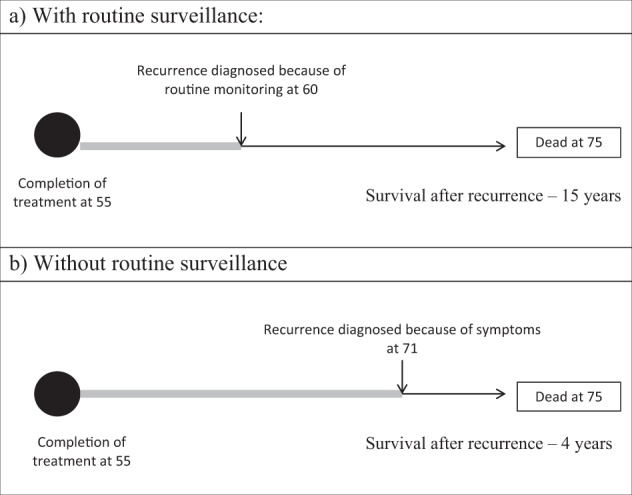
Schematic representation of lead-time bias in cancer recurrence with (**a**) or without (**b**) routine surveillance, adapted from Wegwarth et al. [31]

False positive results and a diagnosis of cancer can also affect a patient’s mental well-being. Patients concerned about thyroid cancer recurrence reported low Health-Related Quality of Life (HRQoL), similar to those who actually had disease recurrence [30]; suggesting that reminder of their previous diagnosis may cause psychological harm. Patients may also be misled by any apparent survival benefit (Fig. 2), as a result of ‘lead time bias’ [31].

The financial burden of routine surveillance accounts for over one-third of all expenditure on DTC in the US [32]. This amounts to almost $600 million and is projected to be as much as $1.4 billion in 2030 [32]. The cost of detection of recurrent disease in the low-risk group was seven times greater than the detection of recurrences in the high-risk group [33]. This is most likely due to the higher frequency of recurrent disease in the latter cohort. In the absence of a proven benefit on survival or quality of life, cost-effectiveness cannot be determined.

Systematic reviews that find no eligible studies are sometimes referred to as ‘empty reviews’ [34]. Postulated reasons include novel research areas and the use of strict inclusion criteria. Although these reviews may be considered to be of limited use [34], they highlight the lack of evidence on interventions that are currently considered to be ‘standard’ practice [35]. Historically, many practices and interventions in medicine have been based on anecdote or on biologically plausible mechanisms and theories in the absence of empirical data. To ensure continuation of adherence to an evidence-based medical paradigm, all interventions in standard practice should be assessed for risks and benefits from good quality data to ensure that decisions are made for the benefit of patients.

Amendments to systematic review protocols to include single arm studies in reviews without RCTs and two-arm observational studies may be viewed as unconventional. Furthermore, by revisiting previously excluded studies, there is potential that the review could be deemed unsystematic and biased [36]. However, this deviation in methods was necessary to outline what the current state of the evidence is and has been noted on the PROSPERO website. Without a control arm to make comparisons, only limited conclusions can be drawn. However, single arm studies provide a source of valuable data on clinically relevant outcomes [35]. They also provide baseline parameters on the basis of which further interventional studies are designed. Good quality single arm studies may also be considered sufficient for rare conditions or uncommon outcomes [37].

The three single arm studies are very heterogenous in terms of risk classification, monitoring protocols, definition of recurrent disease and reporting of outcomes. They however demonstrate low recurrence rates, particularly in ‘low-risk’ patients; irrespective of how risk was defined. A significant proportion of reported recurrences was not detected by Tg. Importantly, these studies do not demonstrate that detection and treatment of clinically asymptomatic recurrence has any influence on survival or quality of life.

The benefits of routine surveillance in other areas of oncology have undergone scrutiny. In breast cancer, rigorous and lifelong follow up has no beneficial effect on survival and despite increased identification of recurrence, management was not significantly affected [38]. In colorectal cancer, no statistically significant effect on overall survival, cancer-specific survival or relapse-free survival has been found with increase in the intensity of surveillance [39].

The lack of original data from RCTs and observational studies is a significant limitation of the evidence base. However, there are practical issues in performing RCTs including potential ethical concerns regarding equipoise between intervention and control arms, delayed occurrence and uncommon nature of relevant end-points (such as recurrence, thyroid cancer-specific mortality and overall mortality); the latter necessitating large sample sizes and long follow-up periods that are often unrealistic in large RCTs. Although unlikely, it is possible that eligible studies in non-English language literature may have been missed. It is also a limitation that inclusion criteria was revised to allow the assessment of initially excluded single arm studies; however, this was deemed necessary in light of the paucity of higher quality studies.The eligible studies did not confirm recurrence by histology, which may limit the validity of their results. These studies have not differentiated between differentiated tumours of the papillary, follicular and hurthle cell variety. The potential differences in the biologic behaviour of these tumour types and subtypes [40, 41] should prompt independent scrutiny of the utility of follow-up interventions in each of these subtypes; however, this may be difficult given the uncommon nature of follicular and hurthle cell types.

In summary, international guidelines and recommended current practice in the follow-up of patients with DTC are based on low-quality evidence. There therefore is a need for re-evaluation of current practice and consideration of the need for routine follow up, particularly in patients with low-risk thyroid cancer.
